# Evaluation of the Antioxidant Capacities and Cytotoxic Effects of Ten* Parmeliaceae* Lichen Species

**DOI:** 10.1155/2016/3169751

**Published:** 2016-12-18

**Authors:** C. Fernández-Moriano, E. González-Burgos, P. K. Divakar, A. Crespo, M. P. Gómez-Serranillos

**Affiliations:** ^1^Department of Pharmacology, Faculty of Pharmacy, University Complutense of Madrid, Plaza Ramón y Cajal s/n, 28040 Madrid, Spain; ^2^Department of Plant Biology II, Faculty of Pharmacy, University Complutense of Madrid, Plaza Ramón y Cajal s/n, 28040 Madrid, Spain

## Abstract

Parmeliaceae represents the largest and widespread family of lichens and includes species that attract much interest regarding pharmacological activities, due to their production of unique secondary metabolites. The current work aimed to investigate the* in vitro* antioxidant and cytotoxic activities of the methanol extracts of ten Parmeliaceae species, collected in different continents. Methanol extraction afforded high phenolic content in the extracts. The antioxidant activity displayed by lichens was evaluated through chemical assays, such as the ORAC (Oxygen Radical Absorbance Capacity) and 1,1-diphenyl-2-picrylhydrazyl (DPPH) radical scavenging activities and the ferric reducing antioxidant power (FRAP). A moderately positive correlation was found between the phenolic content and the antioxidant properties for all the species: *R*: 0.7430 versus ORAC values, *R*: 0.7457 versus DPPH scavenging capacity, and *R*: 0.7056 versus FRAP reducing power. The methanol extract of* Flavoparmelia euplecta* exhibited the highest ORAC value, the extract of* Myelochroa irrugans* showed the maximum DPPH scavenging capacity, and* Hypotrachyna cirrhata* methanol extract demonstrated the highest reducing power. Further, the cytotoxic activity of the ten species was investigated on the human cancer cell lines HepG2 and MCF-7;* Myelochroa irrugans* exhibited the highest anticancer potential. The pharmacological activities shown here could be attributed to their phytochemical constituents.

## 1. Introduction

Lichens are generally defined as symbiotic organisms resulting from the successful association between a fungus (the mycobiont) and an extracellularly located photosynthetic partner (the photobiont), which in most cases is represented by green algae, but it can also be replaced by cyanobacteria (10% of lichen symbiosis) or by the simultaneous association of both algae and cyanobacteria (3-4%) [1]. The number of lichen species described so far (more than 27000) varies depending on authors criteria and is in continuous change due to the inclusion of molecular data [2]. They are all characterized by their capacity to survive in the most adverse and diverse geoclimatic circumstances; actually, lichens are found disseminated from the poles to the tropics and from the highest mountains to the plains on earth and substrates, which is mainly favored by their evolved strategy of poikilohydry [3].

Most of the lichenized fungi belong to the phylum Ascomycota (98% of lichen species) and, among them, Parmeliaceae (Ascomycota, Lecanorales) represents the largest family of lichens. This family is widely distributed in different latitudes of both Northern and Southern hemispheres and is remarkably the best studied from systematic and phylogenetic perspectives [4]. Lichen species included in Parmeliaceae (ca. 2700 species grouped in 80 genera) are supposedly around the 10% of total lichen species and they are characterized by having cupulate exciple, foliose, dorsiventral, and rhizinate lower surface, fruticose to subfruticose threadlike thallus, and a gray, yellow-green, and brown to olive-brown upper surface. Parmeliaceae includes several commonly known groups of lichens such as the Iceland moss (*Cetraria islandica*), beard-lichens (*Usnea* sp.), and the Oakmoss (*Evernia prunastri*) [5].

Those and other species of lichens have been used throughout ages with various purposes, in particular as dyes, perfumes, bioindicators of air pollutants, and medicinal remedies in folk medicines. As examples of therapeutic uses,* Usnea barbata* was used to treat hair-related diseases,* Parmelia sulcata* for cranial maladies, and* Parmelia saxatilis* for the treatment of epilepsy [6]. However, in comparison to other natural products, biological activities of lichens are poorly known and its thorough research is mainly being developed in the last two decades; Parmeliaceae arises as the family with the highest pharmacological potential [7]. In general, the pharmacological interest of lichens relies on the capacity of the mycobiont to produce secondary metabolites, which differ from those found in nonlichenized fungi. These unique compounds normally play an adaptive role in the symbiosis, with functions including the regulation of cell division of photobionts, allelopathy, antiherbivory, chelation of heavy metals, and light screening [8]. Besides, lichen metabolites and especially the polymalonyl derived polyketides (such as depsides and depsidones) have been found to exhibit manifold biological activities with potential application in human pharmacology [9, 10]. Various scientific reports suggested that lichens present antimicrobial, antiprotozoal, anti-inflammatory, antipyretic, and antiproliferative (antitumor) activities [11–13]. Regarding their antioxidant potential, it could be considered that lichens are poorly known when compared to higher plants or other fungus, despite recent investigations are dealing with the issue; only a few lichens species and compounds have exerted promising antioxidative potentials (as reviewed in Fernández-Moriano et al. [14]) and further studies are encouraged to deeply understand the value of lichen compounds as protective antioxidant agents.

Antioxidants comprise a heterogeneous group of compounds that share common actions in the oxidation process, such as stopping, retarding, or preventing the effects mediated by the reactive species derived from oxygen (ROS) or nitrogen (RSN) towards oxidizable substrates in biological systems. Antioxidants are especially relevant if one considers that numerous physiological and pathological processes in the human cells produce free radicals and reactive species. What is more, their overproduction leads to a situation of cellular oxidative stress, in which the endogenous antioxidant systems cannot overcome the damaging effect of ROS such as superoxide anion (O^2−^), hydrogen peroxide (H_2_O_2_), hydroxyl radical (HO^*∙*^), and singlet oxygen (^1^O_2_) [15]. Therefore, oxidative stress involves the damage to biomolecules (DNA, membrane lipids, and enzymes, among others) and is eventually a pathogenic feature of chronic diseases, including cancer and age-related neurodegenerative diseases [16, 17]. It takes special relevance within the brain cells, as they are extremely susceptible to oxidative stress and ROS due to their major consumption of oxygen and the higher content of polyunsaturated fatty acid (prone to peroxidation), among other reasons [18].

Many authors agree with the idea that one of the most efficient ways to counteract oxidative stress-mediated cytotoxicity is through exogenous antioxidant supplementation; antioxidant compounds may act through different mechanisms including scavenging of ROS, induction of endogenous antioxidant enzymes, and chelation of excess catalytic metals (iron, copper). Since several synthetic antioxidants have demonstrated toxicity in humans (e.g., BHA, BHT), consistence evidences support the research and use of antioxidant compounds of natural origin with that aim. What is more, many natural antioxidants, such as flavonoids, resveratrol, and other polyphenols, have been proposed as potential therapeutic tools for the prevention or delay of neurodegenerative diseases (e.g., Alzheimer's and Parkinson's disease), which currently lack effective treatments [19–21]. Consequently, in a context of growing interest towards the finding of antioxidant compounds from plant resources without any undesirable effect, in the last few years lichens emerge as an attractive field of research.

In view of this information and as part of our present research, herein we report a screening on the antioxidant capacity and phenolic profile of the methanol extract of ten Parmeliaceae lichen species from diverse locations. Out of all lichen species under investigation, some of the selected species have already been investigated for antimicrobial or enzyme inhibitory activities [22, 23], but little is known about their antioxidant capacities. In addition, as an approach to their anticancer potential, we aimed to determine their cytotoxic effects on two human cancer cell lines (the hepatocellular carcinoma-derived HepG2 and the breast adenocarcinoma MCF-7 cell line).

## 2. Materials and Methods

### 2.1. Chemicals

RPMI 1640 medium, fetal bovine serum (FBS), phosphate-buffered saline (PBS), and gentamicin were obtained from Gibco (Invitrogen, Paisley, UK). 3-(4,5-Dimethyl-2-thiazolyl)-2,5-diphenyltetrazolium bromide (MTT) and dimethyl sulphoxide (DMSO), 1,1-diphenyl-2-picrylhydrazyl (DPPH), 2,2′-azobis(2-methylpropionamidine)-dihydrochloride (AAPH), fluorescein disodium salt, and 2,4,6-Tris (2-pyridyl)-1,3,5-triazine (TPTZ) were provided by Sigma-Aldrich (St. Louis, MO, USA).

### 2.2. Lichen Samples

The investigated lichen samples were collected, identified, and authenticated by a taxonomist and the voucher specimens are preserved in the lichen section of MAF herbarium, Faculty of Pharmacy, Universidad Complutense de Madrid, Madrid, Spain (MAF-Lich). Their identifying data are as follows:

- *Bulbothrix setschwanensis* (Zahlbr.) Hale, Uttarakhand, Uttarkashi district, India, November 2012, MAF-LICH 20660.
- *Flavoparmelia caperata* (L.) Hale, Gran Canaria, Canary Islands, Spain, June 2009, MAF-LICH 20662.
- *Flavoparmelia euplecta* (Stirt.) Hale, New South Wales, Australia, February 2004, MAF-LICH 15375.
- *Flavoparmelia haysomii* (CW Dodge) Hale, Canberra, Australia, September 1999, MAF-LICH 7535.
- *Hypotrachyna cirrhata* (Fr.) Hale, Uttarakhand, Uttarkashi district, Kedarkantha, India, November 2012, MAF-LICH 20659.
- *Lethariella canariensis* (Ach.) Krog, Madeira, Portugal, September 2012, MAF-LICH 20663.
- *Myelochroa irrugans* (Nyl.) Elix & Hale, Chichibu city, Nakatsugawa, Prefecture Saitama, Province Musashi, Honshu, Japan, February 2009, MAF-LICH 303.
- *Parmelia omphalodes* (L.) Ach, Candeleda, Castilla y León, Spain, July 2015, MAF-LICH 20661.
- *Usnea aurantiacoatra* (Jacq.) Bory, Navarino, La Bandera, Chile, January 2008, MAF-LICH 15686.
- *Usnea contexta* Motyka, Navarino, La Bandera, Chile, January 2005, MAF-LICH 15710.

### 2.3. Lichen Extracts Preparation

Dry thalli of the investigated lichens (50 mg) were extracted in methanol (2 ml) for 2 h, by the extraction method of shaking maceration. Every 30 min, flasks were shaken in vortex for 1 min. The extraction was performed at room temperature (20–22°C). Afterwards, extracts were filtered (through nylon filters of 0.45 *μ*m pore) and then evaporated to dryness at room temperature [24]. The dry residues were then weighted and kept at 4°C. Finally, extraction yields were calculated as the percentage of air-dried weight lichens/weight of the original thallus sample.

### 2.4. Antioxidant Activities

#### 2.4.1. ORAC Assay

The ORAC assay was performed as previously described by Dávalos et al. [25]. Trolox was used as the antioxidant reference compound. The samples were dissolved at a concentration of 1 mg/ml in methanol and then progressively diluted so that the concentrations in the wells ranged from 10 and 500 *μ*g/ml. Lichen extract solutions were incubated with fluorescein (70 nM) for 10 min at 37°C in 96-well plates. After incubation, AAPH (12 mM) was added and fluorescence was recorded for 98 min at excitation and emission wavelengths of 485 nm and emission of 520 nm, respectively, in a FLUOstar Optima fluorimeter (BMG Labtech, Ortenberg, Germany). Results are expressed as *μ*mol Trolox equivalents (TE)/mg sample.

#### 2.4.2. DPPH Assay

The DPPH assay was done according to the method described by Amarowicz et al. [26] with some modifications. Lichen extracts solutions were incubated with DPPH (50 *μ*m) for 30 min at dark in 96-well plates. Absorbance was then measured at 517 nm in a FLUOstar Optima fluorimeter (BMG Labtech, Ortenberg, Germany). Trolox was used as antioxidant reference compound. Results are expressed as EC_50_ value (effective concentration in *μ*g/ml).

#### 2.4.3. FRAP Assay

The ferric reducing antioxidant activity of lichen extracts was measured using FRAP assay by the method described Sánchez-Muniz et al. [27]. The working FRAP reagent was prepared by mixing TPTZ (2,4,6-tri(2-pyridyl)-s-triazine), ferric chloride, and buffer solution. Lichen extracts (1 mg/ml) were then mixed with working FRAP reagent and incubated for 30 min at 37°C. Absorbance was read at 595 nm in a Spectrostar Nanomicroplate reader (BMG Labtech Inc., Ortenberg, Germany). The values are expressed as *μ*mol Fe^2+^/g extract.

### 2.5. Determination of Total Phenolic Content

The total phenolic content in lichen extracts was measured using the Folin-Ciocalteu assay [28]. Briefly, lichen extract at 1 mg/ml in methanol (0.5 ml) was added to test tubes and mixed with Folin-Ciocalteu reagent (0.5 ml), Na_2_CO_3_ solution (75 g/l; 10 ml), and distilled water (14 ml). This reaction mixture was then incubated at dark for 1 h. Absorbance was measured at 760 nm using a spectrophotometer (Uvikon930, Kontron Instruments, Bardsey, UK) and compared to a gallic acid calibration curve. Results are expressed as *μ*g gallic acid equivalent/mg dry extract.

### 2.6. Cancer Cell Lines

MCF-7 human breast cancer cells and HepG2 human liver cancer lines were obtained from the NCI-Frederick Cancer DCTD Tumor/Cell line Repository (Frederick National Laboratory for Cancer Research, National Cancer Institute). MCF-7 and HepG2 cells were cultured in RPMI 1640 medium, supplemented with FBS (10%), and gentamicin (0.5%) at 37°C in a humidified 5% CO_2_ incubator.

### 2.7. Cell Viability Assay

Cell viability was determined by using the colorimetric MTT metabolic activity assay. Cells were treated with different concentrations of lichen extracts from 5 *μ*g/ml to 800 *μ*g/ml for 24 h. After cell treatments, MTT solution (2 mg/ml) was added and incubated for 1 h at 37°C. Following, MTT was removed and the formazan crystals were dissolved in DMSO. The absorbance was read at 550 nm in a Spectrostar Nanomicroplate reader (BMG Labtech Inc., Ortenberg, Germany) and results were expressed as percentage of cell viability (control absorbance values were taken as 100%) [29].

### 2.8. Statistical Analysis

Data were analyzed by one-way analysis of variance (ANOVA) followed by Tukey's test (*p* < 0.05) for multiple comparisons using Statgraphics Centurion XVI software. Data of all assays in the current work correspond to biologically independent experiments performed in triplicate, and the mean values are shown.

Pearson's correlation coefficient (*R*) has been used to measure the strength and direction of a linear correlation between phenolic content and the different antioxidant activities (in ORAC, DPPH and FRAP assays). Correlations were also calculated using the Statgraphics software. The correlation coefficients are significant at the 0.05 level.

## 3. Results and Discussion

### 3.1. Morphoanatomical Features of the Identified Parmeliaceae Lichens

**Figure 1:**
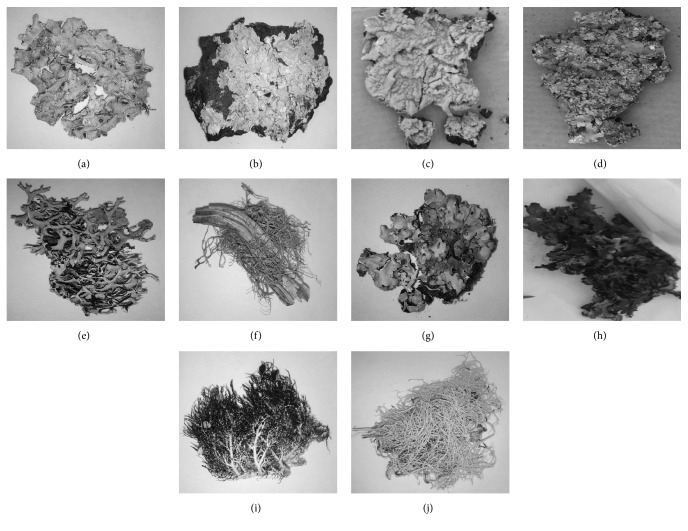
(a)* Bulbothrix setschwanensis* (Zahlbr.) Hale; (b)* Flavoparmelia caperata* (L.) Hale; (c)* Flavoparmelia euplecta* (Stirt.) Hale; (d)* Flavoparmelia haysomii* (CW Dodge) Hale; (e)* Hypotrachyna cirrhata* (Fr.) Hale; (f)* Lethariella canariensis* (Ach.) Krog; (g)* Myelochroa irrugans* (Nyl.) Elix & Hale; (h)* Parmelia omphalodes* (L.) Ach; (i)* Usnea aurantiacoatra* (Jacq.) Bory; (j)* Usnea contexta* Motyka.

The illustrations of the thallus structures of the ten lichen species from Parmeliaceae family investigated in the present study are shown in Figure 1. The selection of these species was based on a geographic distribution pattern, with samples from 4 different continents (Oceania, Asia, Europe, including the Canary Islands, and America), on the presence of diverse extrolites contained in these species and focused on the phytochemistry, pharmacological potential, and phylogenetic features of Parmeliaceae (see [7]).

### 3.2. Extraction Yields and Total Phenolic Content

The results for extraction yields and the content in phenolic compounds of the methanol extracts of the studied species are summarized in Table 1. Yield values ranged between 2.17 and 14.31% w/w. The highest yield was observed for* Myelochroa irrugans* methanol extract, whereas* Usnea aurantiacoatra* methanol extract showed the lowest yield. These extraction yields were similar to those previously reported for the methanol extracts of other Parmeliaceae species [24, 30].

Lichens synthesize unique secondary metabolites, especially depsides, depsidones, and dibenzofuran derivatives with phenol groups in their structure and mainly through the acetyl polymalonyl biosynthetic pathway [9]. Phenolic compounds possess remarkable antioxidant capabilities through free radical scavenging activity, metal ion-chelating action, and modulation of cytoprotective enzymes activity [31]. The total phenolic content was determined by Folin-Ciocalteu method and using a standard curve of gallic acid (0–400 *μ*g/ml).* Flavoparmelia euplecta* showed the highest phenolic content (101.4 *μ*g GA/mg), followed by* Myelochroa irrugans* (92.5 *μ*g GA/mg) and* Parmelia omphalodes* (65.0 *μ*g GA/mg). The lowest phenolic content was found for* Usnea contexta* (20.7 *μ*g GA/mg) and* Usnea aurantiacoatra* (22.4 *μ*g GA/mg). In general, since methanol is used as an efficient solvent for the extraction of phenolic compounds, there is a good correlation between the yield of the maceration process and the content of phenolic compounds [32]. Our results are in line with this:* Usnea* sp. (*U. contexta* and* U. auranticoatra*) showed the lowest yield and phenolic content, whereas* Flavoparmelia euplecta* and* Myelochroa irrugans* were two of the species showing the highest extraction yields.

### 3.3. Antioxidant Capacities

Antioxidant capacities of the ten Parmeliaceae species were determined by evaluating the free radical scavenging activities (ORAC and DPPH assays) and the ferric reducing power (FRAP assay) of their methanol extracts. Results are shown in Table 1.

Through the ORAC assay, we aimed to determine the capacity of lichen extracts to scavenge peroxyl radicals* in vitro*. The ORAC values obtained showed the highest scavenging capacity for* Flavoparmelia euplecta* (3.30 *μ*mol TE/mg dry extract) followed by* Parmelia omphalodes* (3.15 *μ*mol TE/mg dry extract),* Flavoparmelia caperata* (2.81 *μ*mol TE/mg dry extract), and* Myelochroa irrugans* (2.64 *μ*mol TE/mg dry extract). In this assay, the lowest scavenging action was shown for* Usnea aurantiacoatra*, with an ORAC value of 0.32 *μ*mol TE/mg dry extract.

With regard to DPPH method,* Myelochroa irrugans* (EC_50_ = 384 *μ*g/ml) and* Flavoparmelia euplecta* (EC_50_ = 582 *μ*g/ml) presented the strongest DPPH radical scavenging activity. On the other hand, the lowest free radical scavenging effectiveness was displayed by the methanol extract of* Flavoparmelia caperata* (EC_50_ = 3216 *μ*g/ml) and* Lethariella canariensis* (EC_50_ = 2894 *μ*g/ml).

Both ORAC and DPPH assays measure the radical scavenging ability of test samples to neutralize the reactive and oxidative action of free radicals. However, the fundament regarding the mechanisms of antioxidation is different: whereas ORAC method evaluates the antioxidant ability based on the hydrogen atom transference capacity (HAT mechanism), the reaction mechanism of DPPH test proceeds via single electron transfer (ET). These different mechanisms of quenching radicals along the experiments may explain the distinct behaviors of the methanol extracts of the studied Parmeliaceae lichens [33].

FRAP assay measures the reducing power of samples via direct electron donation and the reduction of ferric tripyridyltriazine [Fe^3+^-TPTZ] complex to ferrous tripyridyltriazine [Fe^2+^-TPTZ]. Among the ten methanol extracts studied, the ferric reducing antioxidant power was the highest for* Hypotrachyna cirrhata* (316 *μ*mol of Fe^2+^ eq/g sample),* Flavoparmelia euplecta* (273 *μ*mol of Fe^2+^ eq/g sample), and* Myelochroa irrugans* (266 *μ*mol of Fe^2+^ eq/g sample). In the contrary,* Usnea aurantiacoatra* methanol extract possessed the lowest reducing ferric capacity (98 *μ*mol of Fe^2+^ eq/g sample).

### 3.4. Correlation between Total Phenolic Content and Antioxidant Capacities

Since lichens contain diverse phenolic constituents and these kinds of compounds are known to possess antioxidant properties, correlations between each antioxidant assay (ORAC, DPPH and FRAP) and total phenolic content were investigated by a regression analysis (correlation coefficient, *R*).

**Figure 2:**
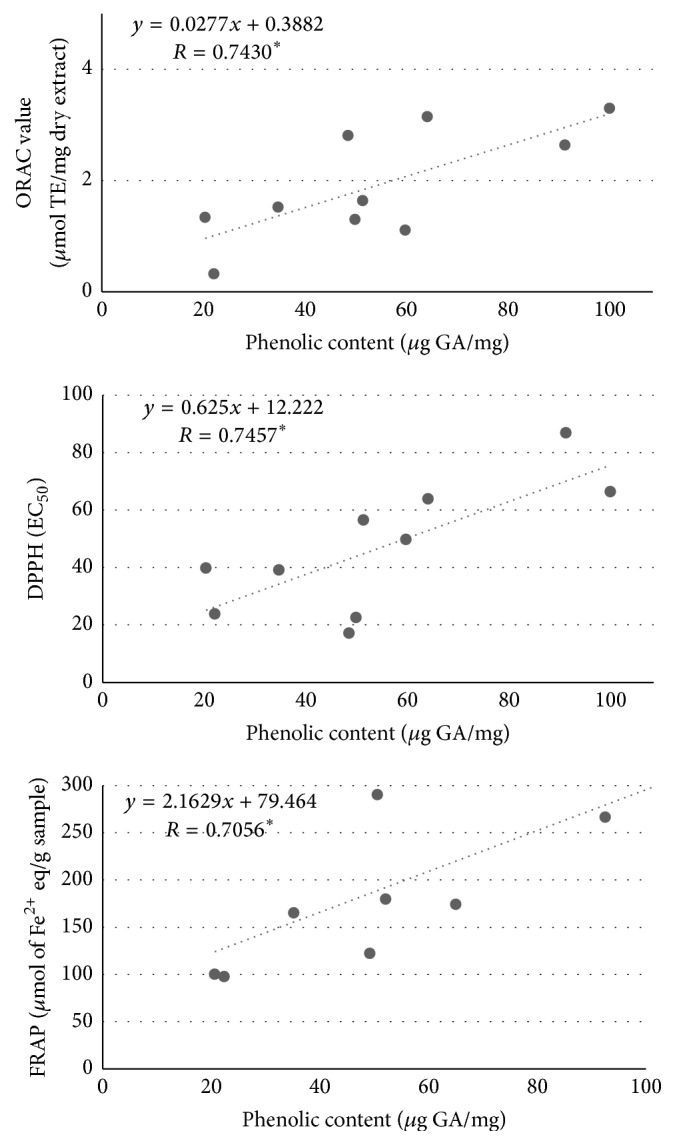
Correlations between each antioxidant assay (ORAC, DPPH, and FRAP) and the total phenolic content. The correlation coefficients are significant at the level of *p* < 0.05.

As shown in Figure 2, there is a moderate to high positive correlation between the antioxidant parameters measured and the amount of phenolic compounds present in the different extracts. Actually, the correlations were very similar for all assays, with the following *R* values versus total phenolic content: *R* = 0.743^*∗*^ for ORAC assay (*R* ^2^ = 0.552); *R* = 0.746^*∗*^ for DPPH assay (*R* ^2^ = 0.556); and *R* = 0.706^*∗*^ for FRAP method (*R* ^2^ = 0.498) (*∗* stands for statistical significance of Pearson's correlation coefficient at *p* < 0.05).

Although slightly higher, ORAC and DPPH radical scavenging capacities presented a better correlation with phenolic content than ferric reducing activity. In general, we found that the lichen extracts showing the highest phenolic content (e.g.,* Flavoparmelia euplecta* and* Myelochroa irrugans*) were among the most active ones in the chemical tests evaluating antioxidant potential via radical scavenging mechanism; also, those with the lowest contents in polyphenols (*Usnea auranticoatra* and* Usnea contexta*) displayed the weakest antioxidant activities. However, we found moderate variations in the effect displayed by other species with intermediate phenolic content.

The lack of a higher correlation between the antioxidant capacities of lichen methanol extracts and the content in polyphenols might be explained by several facts including the structural diversity of phenolic compounds. Previous studies on structure-activity relationship have demonstrated that antioxidant potency and reaction mechanisms of phenol derivatives compounds depend on both hydroxyl group position and number. Lopes et al. [34] confirmed that hydrogen bonding of carbonyl groups to hydroxyl radicals reduced significantly the scavenging activity of lichen constituents. In higher plants, there is scientific consensus on a good correlation between the total phenolic content and the antioxidant capacity of a certain sample [35, 36]. However, there are many discrepancies on such correlation in lichen species. Some authors did not find any positive correlation between antioxidant activity and total phenolic content of lichen extracts [37], but in contrast, others described strong relationships between total phenolic and flavonoid contents and the antioxidant effect [38]. Our results are in line with this information and suggest that the antioxidant activity of some tested extracts might be attributable to the presence of nonphenolic compounds. For instance, apart from phenols, phytochemical analysis of lichens has revealed the presence of terpenoids in different Parmeliaceae species [39], and these minor compounds have demonstrated to possess antioxidant properties and therefore to contribute to the total antioxidant activity [40]. Besides, it should be pointed out that individual phenolics may present distinct antioxidant activities in the different chemical tests, and there may be synergistic or antagonistic interactions between phenolics and other compounds like proteins, carbohydrates, and so forth.

### 3.5. Cytotoxic Activities

Up to date, only a few studies have evaluated the cytotoxic properties of Parmeliaceae lichens. Mitrović et al. [41] reported the antiproliferative activity of the methanol extracts of some Parmeliaceae species different to these investigated in the present study (such as* Parmelia sulcata*,* Flavoparmelia caperata*,* Evernia prunastri*, and* Hypogymnia physodes*) against the colon cancer adenocarcinoma cell line HCT-116. Moreover, Bézivin et al. [42] studied the anticancer activity of eight extracts obtained from Parmeliaceae spp. collected in various places of Brittany, among which* Parmelia caperata*,* Parmelia perlata*, and* Usnea rubicunda* are found, on human and murine cancer lines. However, to our knowledge, the species included in our study has not been previously evaluated against the MCF-7 and HepG2 cell lines.

**Figure 3:**
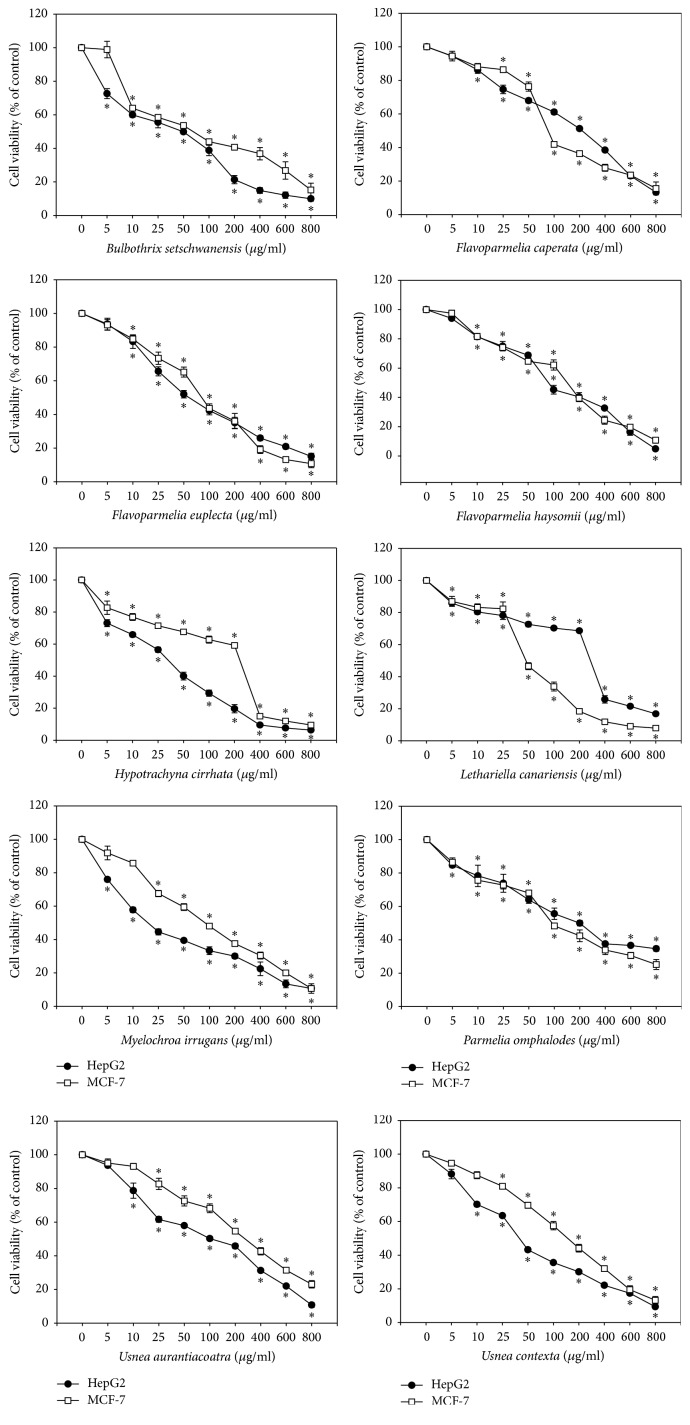
Cell viability of HepG2 and MCF-7 cells treated with different concentrations of Parmeliaceae lichen extracts for 24 h. Cell viability of control cells was normalized to 100%. Means values ± SD, ^*∗*^ *p* < 0.05 versus control.

Therefore, the cytotoxic effects of the methanol extracts of the ten studied Parmeliaceae species were evaluated, as an approach to their anticancer potential, against the human-derived MCF-7 (human breast adenocarcinoma) and HepG2 (human hepatocellular carcinoma) cancer cell lines. Their effects on cell viability were analyzed and quantified by using MTT assay after 24-hour treatment with a range of concentrations of extracts from 5 to 800 *μ*g/mL. Through this assay, cell viability is determined based on the measurement of mitochondrial function, as MTT is transformed into formazan crystals in living cells in which mitochondrial dehydrogenases are functional. As shown in Figure 3, cell viability decreased in a concentration-dependent manner in both MCF-7 and HepG2 cell lines for all assayed lichen extracts. The lethal doses 50% (LD_50_) were determined and these data are shown in Table 2. Differences among cancer cell lines types in their sensitivity to tested methanol lichen extracts were observed.

The lichen extracts that possessed stronger cytotoxic activity towards MCF-7 human breast cancer cells were* Lethariella canariensis* (LD_50_ = 66 *μ*g/mL) followed by* Flavoparmelia euplecta* (LD_50_ = 67 *μ*g/mL) and* Bulbothrix setschwanensis* (LD_50_ = 91 *μ*g/mL). In contrast,* Usnea aurantiacoatra, Hypotrachyna cirrhata*, and* Flavoparmelia haysomii* exhibited very low toxicity with LD_50_ values of 339 *μ*g/mL, 281 *μ*g/mL, and 212 *μ*g/mL, respectively. The screening for cytotoxicity against HepG2 human hepatocarcinoma cells revealed that* Myelochroa irrugans* reduced significantly their cell viability with LD_50_ of 22 *μ*g/mL; methanol extracts of the Parmeliaceae species* Hypotrachyna cirrhata* (LD_50_ = 41 *μ*g/mL) and* Usnea contexta* (LD_50_ = 54 *μ*g/mL) induced also remarkable toxicity against HepG2 cells. On the other hand, the less active lichen extracts were* Lethariella canariensis* and* Flavoparmelia caperata* which showed values of LD_50_ of 351 *μ*g/mL and 272 *μ*g/mL, respectively. According to the National Cancer Institute from USA, crude extracts are considered to possess significant cytotoxic activity when IC_50_ < 30 *μ*g/mL. The methanol extract of* Myelochroa irrugans* falls within this criteria, being of interest as anticancer agent against liver cancer.

Currently there is growing evidence that supports the involvement of reactive oxygen species in the etiology of fat-related neoplasms such as cancer of the breast, colorectum, and liver due to the peroxidation of lipids and subsequent formation of toxic aldehydes [43]. With this regard, antioxidant phenolic compounds may counteract the carcinogenic potential of ROS via scavenging of free radicals and blockage of peroxidation chain reaction. Actually, positive correlation between phenolic content and cytotoxic activities against human cancer cell lines has been found* in vitro* [44, 45]. This correlation may explain the interesting cytotoxic effects displayed by the methanol extracts of* Myelochroa irrugans* and* Flavoparmelia euplecta*.

Based on the overall results for antioxidant and cytotoxic activities, the methanol extracts of the three species that exerted the most promising pharmacological potential were* Flavoparmelia euplecta*,* Myelochroa irrugans*, and* Parmelia omphalodes*. These three species belong to the major phylogenetic clade in the Parmeliaceae family: the parmelioid clade. Within the parmelioid clade,* Flavoparmelia* is grouped in the parmotremoid clade whereas* Myelochroa* and* Parmelia* belong to the* Parmelina* and* Parmelia* clades, respectively [46].

For these lichen species, there is not any previous study that has evaluated their antioxidant or their cytotoxic/anticancer actions. The biological properties demonstrated herein can be attributed to the presence of bioactive secondary metabolites in the extracts.* Flavoparmelia euplecta* has been reported to contain the dibenzofuran usnic acid and the depsidone protocetraric acid [47]; on the other hand,* Myelochroa irrugans* present as bioactive compounds the depside atranorin, the triterpenes zeorin and leucotylic acid, and the xanthone secalonic acid A [48]; and the main secondary metabolites in* Parmelia omphalodes* have been described to be the depsides atranorin, the depsidones salazinic and lobaric acids, and the acyclic fatty acid protolichesterinic acid [49].

There are some previous studies that have investigated both the antioxidant and the cytotoxic activities of some of these lichen substances, and their results may help to explain our findings. Usnic acid has been one of the most studied lichen compound for its pharmacotoxicological actions, including its antioxidant potential. This dibenzofuran has shown to increase the levels of superoxide dismutase (SOD), glutathione peroxidase (GPx), and reduced glutathione (GSH) and to reduce lipid peroxidation on indomethacin-induced gastric ulcer in rats [50]. In another study, usnic acid and also salazinic acid (although to a lesser extent) have demonstrated protecting astrocytes against hydrogen peroxide-induced oxidative stress by increasing cell viability and inhibiting intracellular ROS production; this protection seems to be related, at least in part, to its peroxyl scavenger properties [51]. Moreover, usnic acid was also effective as an antioxidant agent against lipopolysaccharide-induced lung injury through the decrease of the levels of hydrogen peroxide, myeloperoxidase, and malondialdehyde (MDA), and the increase of the levels of superoxide dismutase (SOD) and reduced glutathione (GSH) [52]. Atranorin has also shown to possess remarkable antioxidant activity by inhibiting lipid peroxidation [53] and acting as a superoxide [54] and free radical diphenyl picryl hydrazyl (DPPH^*∗*^) scavenger [55]. Manojlović et al., [56] evidenced that salazinic acid has stronger superoxide anion radical scavenging activity and reducing power than protocetraric acid.

With respect to cytotoxic/anticancer activities, the isolated compounds protocetraric acid and usnic acid have demonstrated to significantly inhibit the growth of the melanoma UACC-62 and B16-F10 cell lines [56]. In another study, the compounds salazinic acid and protocetraric acid were proved to exert high cytotoxic activity against FemX (human melanoma) and LS174 (human colon carcinoma) [57]. Moreover, atranorin and usnic acid are potent anticancer agents in both the A2780 human ovarian cancer cell line and the HT-29 human colon cancer cell line, acting through a mitochondrial pathway [58]. Furthermore, the compound protolichesterinic acid has resulted to be a promising agent against the cervix adenocarcinoma HeLa cell line via activation of caspase pathway [59].

Still, further studies are required to better understand the antioxidant and cytotoxic potentials of the lichen species studied here and their active metabolites. It would be of interest to isolate the main bioactive compounds found in* Flavoparmelia euplecta*,* Myelochroa irrugans,* and* Parmelia omphalodes* and to determine which compound contributes in a higher degree to the antioxidant activity and cytotoxic action against MCF-7 and HepG2 cell lines.

## 4. Conclusions

Taken together, our results suggest that the Parmeliaceae lichen species* Flavoparmelia euplecta* and* Myelochroa irrugans*, which showed the highest antioxidant and cytotoxic actions, may arise as promising sources of natural compounds with pharmacological interest. Further research is encouraged to determine the real potential of their secondary metabolites in the therapy of oxidative stress-related diseases and cancer.

## Figures and Tables

**Table 1:** Yields of extraction, antioxidant capacities (ORAC method, DPPH assay, and FRAP method), and total phenolic content of the ten methanol extracts of Parmeliaceae lichens species. Statistical significances (of antioxidant capacities values) for multiple comparisons from Tukey's test are shown in superscripts. a: statistically significant differences versus values of *Bulbothrix setschwanensis*; b: versus *Flavoparmelia caperata*; c: versus *Flavoparmelia euplecta*; d: versus *Flavoparmelia haysomii*; e: versus *Hypotrachyna cirrhata*; f: versus *Lethariella canariensis*; g: versus *Myelochroa irrugans*; h: versus *Parmelia omphalodes*; i: versus *Usnea aurantiacoatra*; j: versus *Usnea contexta* (*p* < 0.05).

Lichen species	Yield	ORAC value	DPPH EC_50_	FRAP	Phenolic content
	(% w/w)	(*μ*mol TE/mg dry extract)	(*μ*g/mL)	(*μ*mol of Fe^2+^ eq/g sample)	(*μ*g GA/mg)
*Bulbothrix setschwanensis*	9.83 ± 1.68	1.64 ± 0.13^b,c,e,g,h,i^	851^b,c,d,f,g,i,j^	180 ± 3^b,c,e,f,g,i,j^	52.2 ± 0.2
*Flavoparmelia caperata*	11.31 ± 1.95	2.81 ± 0.33^a,c,d,e,f,i,j^	3216^a,c,d,e,g,h,i,j^	122 ± 2^a,c,d,e,f,g,h,i,j^	49.2 ± 0.2
*Flavoparmelia euplecta*	12.14 ± 3.36	3.30 ± 0.24^a,b,d,e,f,g,i,j^	582^a,b,d,e,f,i,j^	273 ± 2^a,b,d,e,h,i,j^	101.4 ± 0.3
*Flavoparmelia haysomii*	14.02 ± 3.00	1.52 ± 0.08^b,c,e,g,h,i^	1444^a,b,c,e,f,g,h,i^	165 ± 4^b,c,e,f,g,i,j^	35.2 ± 0.3
*Hypotrachyna cirrhata*	9.70 ± 2.05	1.11 ± 0.07^a,b,c,d,g,h,i^	946^b,c,d,f,g,i^	316 ± 3^a,b,c,d,f,g,h,i,j^	60.6 ± 0.2
*Lethariella canariensis*	5.21 ± 1.31	1.30 ± 0.07^b,c,g,h,i^	2894^a,c,d,e,g,h,i,j^	290 ± 3^a,b,d,e,h,i,j^	50.6 ± 0.2
*Myelochroa irrugans*	14.31 ± 2.80	2.64 ± 0.25^a,c,d,e,f,h,i,j^	384^a,b,d,e,f,i,j^	266 ± 6^a,b,d,e,h,i,j^	92.5 ± 0.3
*Parmelia omphalodes*	7.32 ± 1.22	3.15 ± 0.14^a,d,e,f,g,i,j^	680^b,d,f,i,j^	174 ± 2^b,c,e,f,g,i,j^	65.0 ± 0.2
*Usnea aurantiacoatra*	2.17 ± 0.65	0.32 ± 0.03^a,b,c,d,e,f,g,h,j^	2446^a,b,c,d,f,g,h,j^	98 ± 2^a,c,d,e,f,g,h^	22.4 ± 0.3
*Usnea contexta*	2.64 ± 0.61	1.34 ± 0.15^b,c,g,h,i^	1332^a,b,c,f,g,i^	100 ± 1^b,c,d,e,f,g,h^	20.7 ± 0.2

**Table 2:** Values of LD_50_ for the methanol extracts of the ten studied Parmeliaceae lichens species towards MCF-7 and HepG2 cells in treatments of 24 h.

Lichen species	LD_50_ (*μ*g/ml) MCF-7	LD_50_ (*μ*g/ml) HepG2
*Bulbothrix setschwanensis*	91	92
*Flavoparmelia caperata*	131	272
*Flavoparmelia euplecta*	67	67
*Flavoparmelia haysomii*	212	193
*Hypotrachyna cirrhata*	281	41
*Lethariella canariensis*	66	351
*Myelochroa irrugans*	145	22
*Parmelia omphalodes*	138	260
*Usnea aurantiacoatra*	339	151
*Usnea contexta*	208	54
